# MiR-130a and MiR-374a Function as Novel Regulators of Cisplatin Resistance in Human Ovarian Cancer A2780 Cells

**DOI:** 10.1371/journal.pone.0128886

**Published:** 2015-06-04

**Authors:** Ningwei Li, Lingyun Yang, Hongjing Wang, Tao Yi, Xibiao Jia, Cen Chen, Pan Xu

**Affiliations:** Department of Gynecology and Obstetrics, West China Second University Hospital, Sichuan University, Chengdu, Sichuan, 610041, P.R. China; The University of Hong Kong, CHINA

## Abstract

Chemoresistance remains a major obstacle to effective treatment in patients with ovarian cancer, and recently increasing evidences suggest that miRNAs are involved in drug-resistance. In this study, we investigated the role of miRNAs in regulating cisplatin resistance in ovarian cancer cell line and analyzed their possible mechanisms. We profiled miRNAs differentially expressed in cisplatin-resistant human ovarian cancer cell line A2780/DDP compared with parental A2780 cells using microarray. Four abnormally expressed miRNAs were selected (miR-146a,-130a, -374a and miR-182) for further studies. Their expression were verified by qRT-PCR. MiRNA mimics or inhibitor were transfected into A2780 and A2780/DDP cells and then drug sensitivity was analyzed by MTS array. RT-PCR and Western blot were carried out to examine the alteration of MDR1, PTEN gene expression. A total of 32 miRNAs were found to be differentially expressed in A2780/DDP cells. Among them, miR-146a was down-regulated and miR-130a,-374a,-182 were upregulated in A2780/DDP cells, which was verified by RT-PCR. MiR-130a and miR-374a mimics decreased the sensitivity of A2780 cells to cisplatin, reversely, their inhibitors could resensitize A2780/DDP cells. Furthermore, overexpression of miR-130a could increase the MDR1 mRNA and P-gp levels in A2780 and A2780/DDP cells, whereas knockdown of miR-130a could inhibit MDR1 gene expression and upregulate the PTEN protein expression .In a conclusion, the deregulation of miR-374a and miR-130a may be involved in the development and regulation of cisplatin resistance in ovarian cancer cells. This role of miR-130a may be achieved by regulating the MDR1 and PTEN gene expression.

## Introduction

Ovarian cancer is the leading cause of death in gynecological malignancies [1]. More than 70% patients with ovarian cancer have advanced stage (FIGO stage III or IV) disease at the time of diagnosis [2]. Cisplatin is the main therapeutic approach for advanced ovarian cancer, however, drug-resistance minimizes the effectiveness of cisplatin-base chemotherapy in a large number of patients[3]. Multiple factors and mechanisms contributing to cisplatin- resistance have been reported, including increased drug efflux, abnormity of drug target, enhancement of DNA repair and alteration of apoptosis pathways[4–7].Nonetheless the underlying mechanisms of chemoresistance are still poorly understood and the effective agents to improve the resistance is till in absence.

Recently, studies reported that microRNAs (miRNAs) were involved in chemoresistance. MiRNAs represent a class of small non-coding RNA molecules, and could bind to the 3'-untranslated region (3'-UTR) of the target mRNAs, resulting in RNA degradation and/or translational repression [8]. Thus, miRNAs widely participate in the regulation of various biological processes, such as embryonic development, cell proliferation, differentiation, migration and apoptosis[8,9]. A growing number of studies suggest that aberrant miRNA expressions have been associated with every aspect of tumor biology, including resistance to various chemotherapeutic agents[10–12].For instance, miR-21 was overexpressed in colorectal cancer tissues which were less sensitive to 5-FU[13], and inhibition of miR-21 expression was able to sensitize cells to 5-FU[14]. In addition, our previous study[15] had found that miR-130a was upregulated in SKOV3/DDP compared with SKOV3, and miR-130a inhibition could overcome the cisplatin resistance by regulating the MDR1/P-gp pathway. However, the role of miRNAs prensents cell specificity, so the expression of miR-130a or other miRNAs and their roles in other ovarian cancer cell lines are needed to further study.

In the current study, we screened miRNA expression profile of A2780 and A2780/DDP cells, and selected some of the differentially expressed miRNAs in A2780/DDP for further study. Then, we verify the expression of selected miRNAs by qRT-PCR, and investigated their role in modulating the cisplatin-resistance, which may offer new candidate targets for gene therapy in cisplatin-resistant ovarian cancer.

## Materials and Methods

### Cell culture

The human ovarian cancer cell line A2780 and its cisplatin-resistant subline A2780/DDP were cultured in DMEM medium (Gibco, USA) containing 10% fetal bovine serum (FBS, Gibco, USA), in a humidified incubator with 5% CO_2_ at 37°C. A2780/DPP was alternately fed with medium containing 9 μg/mL cisplatin to maintain drug-resistance and further cultured in drug-free medium for one week before follow-up experiments. Both cell lines were obtained and preserved in Gynecological Oncology of Biotherapy Laboratory, West China Second University Hospital, Sichuan University, Chengdu, China.

### miRNA microarray analysis

Total RNA was extracted from A2780 and A2780/DDP cells using Trizol (Invitrogen, USA) and miRNeasy mini kit (QIAGEN, Denmark) according to manufacturer’s instructions. The quality and quantity were measured by the spectrophotometer (Beckman Coulter, DU730, USA) and RNA integrity was verified by gel electrophoresis. RNA (1ug) was labeled with Hy3 fluorescent using the miRCURY Power labeling kit (Exiqon, Vedbaek, Denmark) and then hybridized on the miRCURYLNA Array (v.18.0, Exiqon) containing 1887 human miRNA capture probes annotated in miRBase 18.0. Following hybridization, the fluorescent signal images were acquired using a Genepix 4000B scanner (Axon Instruments,Union City, CA, USA) and were analyzed with Genepix Pro 6.0 soft-ware (Axon Instruments), in which the median normalization was obtained. Two-fold or larger change was set as a threshold of significant difference.

### Bioinformatics

Potential targets of differentially expressed miRNAs were predicted with the help of PicTar or TargetScan 5.2 software.

### Real-time qRT-PCR

We verified the expression of seleted miRNAs in A2780 and A2780/DDP cells by qRT-PCR. Total RNA of each cell line was extracted and assessed as the above mentioned. MiRNA and U6 cDNA was generated using miRNA specific stem-loop RT primers according to the AMV First Strand cDNA Synthesis Kit (Invitrogen, USA) protocol. All primers were designed and synthesized by Guangzhou RiboBio (RiboBio,China). Real-time qRT-PCR was performed in a 20μl reaction volume on the ABI Stepone plus instrument (USA). Relative miRNA expression was evaluated using the 2^-ΔΔCt^ method and normalized to the expression of U6 small RNA. All qRT-PCRs were performed in triplicates.

### Cell transfection

The miRNA mimic, inhibitor and negative control were designed and chemically synthesized by Guangzhou RiboBio (RiboBio, China). 24h prior to transfection, cells were seeded in 96-well plates with 5000 cells/well and cultured in medium without antibiotics. After cell attachment, transfection was performed using Lipofectamine 2000 (Invitrogen, USA) and Opti-Mem reduced serum media (Invitrogen, Carlsbad, CA) according to the manufacturer’s instructions. The medium was replaced 4-6h after transfection with new fresh medium. The cells were prepared for further analysis 48h after transfection. The efficiency of this liposome-mediated transfection system was dectected with the help of Cy3-siRNA. Cy3-siRNA is a sort of 21-25nt small RNA molecule, similar to miRNA inhibitor or mimics, and can excite fluorescence at the wavelength of 555 nm. The main steps are as follows: cells were seeded into 24-well plates with 10000 cells/well and the transfected with Cy3-siRNA using Lipofectamine 2000 and Opti-Mem medium. 24h after transfection, cells were observed under the fluorescence microscope. The transfection efficiency was presented as the ratio of cells observed under the fluorescence to cells observed under the nomal ligtht. All experiments were repeated three times.

### Cell viability array

After transfection, cells in 96-well plates were exposed to various concentration of cisplatin. Cell proliferation was determined by a colorimetric assay using the CellTiter 96 Aqueous One Solution Cell Proliferation Assay kit (Promega, Madison, WI, USA). After 48h of treatment, 20 ul of One Solution Reagent was added to each well and incubated for 3h at 37°C. The absorbance at 490 nm wavelength was measured using microplate reader (Model 680, Bio-Rad, USA). Each experiment was conducted with replicates of six wells and repeated three times.

### Semi-quantitative RT-PCR

Total RNA from untreated cells or transfected cells was isolated using Trizol Reagent. RT-PCR was performed by using the PrimeScript RT reagent Kit and Multiplex PCR Assay Kit according to the manufacturer's instructions (TaKaRa Biotechnology, Dalian, China). PCR products were identified by electrophoresis with 1.5% agarose gels and recorded using the Gel imaging system (Bio-Rad, CA, USA). GAPDH RNA was served as an input control.

### Western blot assay

The cells were washed with 1×PBS and lysed with RIPA buffer. The protein concentration was measured using the BCA method. Next, 20ug of protein was used for the detection of P-gp and PTEN protein, and GAPDH was used as the loading control. Mouse monoclonal anti-Pgp (diluted 1:1000; Calbiochem, San Diego, CA, USA), anti-PTEN(diluted 1:1000; Santa Cruz, Ca, USA) and anti-GAPDH (diluted 1:50000; Kangchen, Shanghai, China) were used as primary antibodies. The secondary antibody was conjugated with horseradish peroxidase. The bound antibodies were detected using ECL kit. The Quantity One software was used to quantify protein band intensities.

### Statistical analysis

The data are presented as the mean ± SD. The difference between means was analyzed using Student’s t-test. All statistical analyses were performed using SPSS 13.0 software (Chicago, IL, USA). P values less than 0.05 were considered to be statistically significant.

## Results

### Differential expressed miRNAs in A2780/DDP compared to its parental cell A2780

The miRNA expression profile of ovarian cancer cell line A2780s and A2780/DDP was detected using miRNA array in this study. As shown in Table 1, 32 miRNAs (24 up-regulated, 8 down-regulated) were found to be differentially expressed in A2780/DDP cells compared to its parental cells. The potential target gene of these miRNAs was predicted and listed in Table 1.

**Table 1:** 32 differentially expressed miRNAs in A2780/DDP.

miRNA	Fold changes	Predicted targets
hsa-miR-27a-3p	2.01	HCN4, CDH5, SH2D3C, SCAMP3, PPIF
hsa-miR-590-5p	2.06	GNPDA, PDCD6, PTPRU, ATP9A
hsa-miR-1297	2.07	CDH2, NR2E3, KCNE3, TANK,
hsa-miR-196a-3p	2.25	SNX16, AQP4, SLC9A6, EYA4
hsa-miR-374a	2.27	AKT3, KCNE3, PIGK, PDCD6IP
hsa-let-7d-5p	2.29	ABCC5, DPP3, PTPRU, RASGRP, NME6
hsa-miR-1258	2.42	NAALADL, PARP3, IL18BP, TSPAN2
hsa-miR-98	2.44	ABCC5, FARP1, PCGF3, DLC1
hsa-let-7f-5p	2.57	SH2B3, ABCC5, DPP3, RASGRP1
hsa-miR-25-3p	2.59	CDH10, FRY, GDF11, TOB1
hsa-miR-374c-5p	2.62	Unknown
hsa-miR-3651	2.69	Unknown
hsa-miR-130a	2.78	CEBPE, RALBP1,MED12L, PTEN
hsa-miR-128	2.86	GNPDA1, KCNE3, HDAC5,BCL2L10
hsa-miR-3175	3.15	Unknown
hsa-miR-182	3.30	NAMPT, TOB1, MBNL2, FARP1
hsa-miR-31-3p	3.85	ZNF573, UGT3A1, ZFP30, MLXIP
hsa-miR-183-5p	4.89	PDCD6, DUSP10, KIAA0182, IRS1
hsa-miR-378b	7.02	Unknown
hsa-miR-4289	7.06	Unknown
hsa-miR-9-5p	16.66	MARCH6, UBXD8, SIRT1, LIN28B
hsa-miR-200c-3p	17.24	DLC1, CYP1B1, DUSP1, E2F3
hsa-miR-378c	29.32	Unknown
hsa-miR-146b-5p	0.02	NOVA1, STRBP, FBXL10, USP3
hsa-miR-146a-5p	0.01	CCDC117, SFRS6, IRAK1, SMAD4
hsa-miR-155-5p	0.02	JARID2, SYPL1, CHAF1A, AKAP10
hsa-miR-374b-3p	0.50	EDIL3, ACTR2, ADAM10,WDR68
hsa-miR-21-3p	0.41	KCNMB2, SEPT10, PDZD2, SUZ12
hsa-miR-181a-5p	0.10	RNF145,SS18L1, FOXP1, GLS
hsa-miR-1321	0.46	ADARB2,DDX17,TGOLN2, WBP4
hsa-miR-4297	0.30	Unknown

### Real-time qRT-PCR for 4 differentially expressed miRNAs

**Fig 1:**
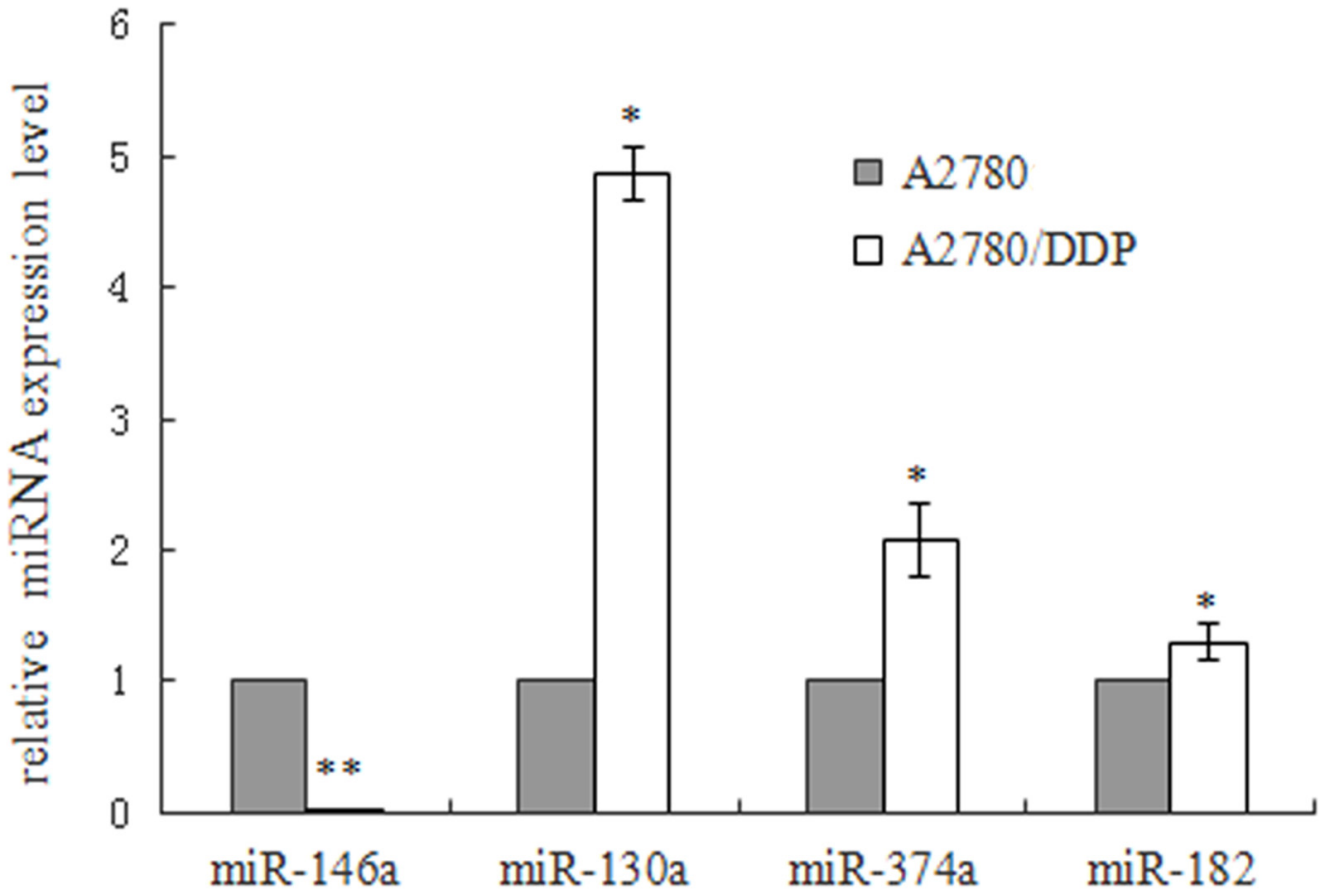
RT-qPCR validation of the selected 4 miRNAs expression in A2780s and A2780/DDP cells. The level of miR-146a in A2780/DDP was extremely low, only accounts for 0.75 percentage of that in A2780s (**p<0.05). The expression of miR-130a, -374a and miR-182 was 4.8, 2.08 and 1.28 folds higher than that in A2780 cells (**p<0.05, *p<0.05).These results showed the consistent expression changes detected by the microarray.

Among 32 differentially expressed miRNAs, we selected 4 miRNAs for futher study. The results of qRT-PCR showed that, in A2780/DDP cells, the expression of miR-374a, 130a, -182 was respectively increased by 2.06, 4.87, 1.28 folds and miR-146a decreased by 133.56 fold compared with A2780s cells(*p*<0.05), which were consistent with the microarray. (Fig 1)

### miR-130a and miR-374a mimics or inhibitors regulate cisplatin sensitivity in A2780 and A2780/DDP

In this study, we used the liposome to mediate the transfection of miRNA analogues. So we firstly detected the transfection efficiency. A2780 and A2780/DDP cells were observed under the fluorescence microscope after 24 hours of Cy3-siRNA transfection. we calculated that the A2780s and A2780/DDP cells with red fluorescence respectively accounted for 85% and 93%, which indictated that Liposome-mediated transfection system could reach a high efficiency.

**Fig 2:**
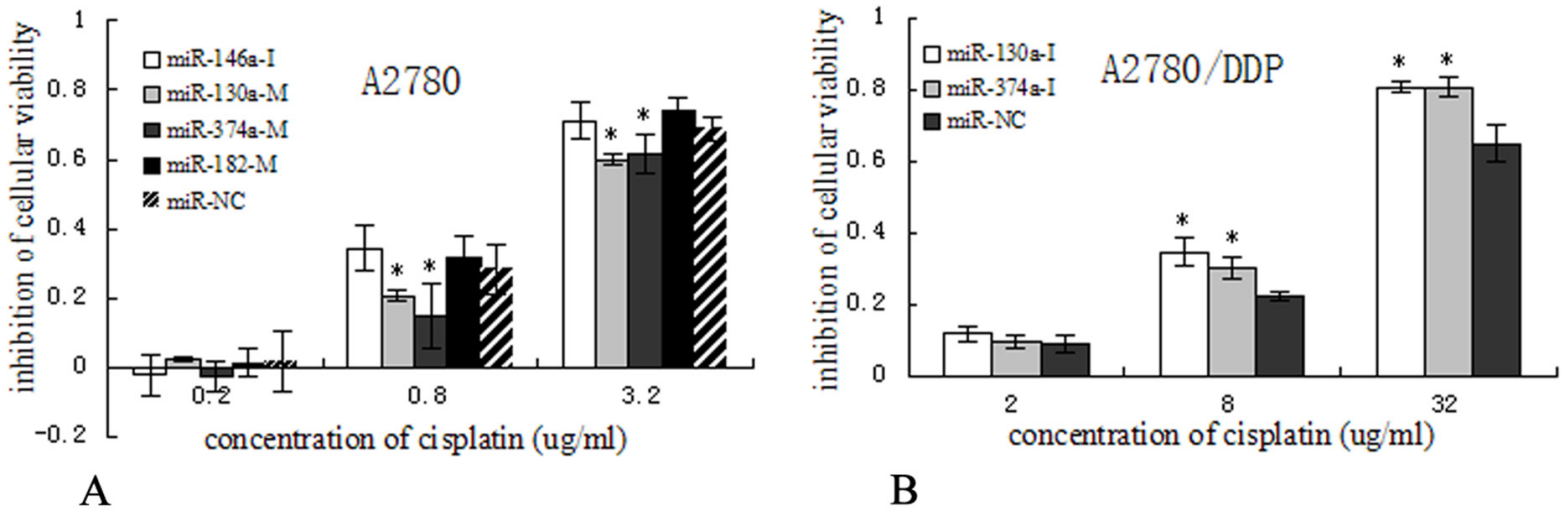
Cells inhibition by cisplatin following pre-treatment with miR-130a, -374a mimics or inhibitors. (A) The A2780 cells after transfection with miR-130a-mimic and miR-374a-mimic showed a increased ratio of surviving cells under the treatment of 0.8, 3.2 ug/ml cispaltin (*p<0.05). (B) miR-130a-inhibitor and miR-374a-inhibitor decreased the ratio of surviving cells under the treatment of 8, 32 ug/ml cispaltin (*p< 0.05).

Three miRNA(miR-374a, -130a, -182) mimics and miR-146a inhibitor were transfected into A2780 cells, to see whether the transfected cells would acquire resistance to cisplatin. The results showed that the A2780 cells transfected with miR-374a and miR-130a mimics had a significantly higher survival than the control group under the treatment of 0.8, 3.2 ug/ml cispaltin, but same phenomenon was not observed for miR-182 mimics and miR-146a inhibitor. (p<0.05, Fig 2A).

We further transfected with miR-374a and miR-130a inhibitors into A2780/DDP cells to explore whether they can partially reverse the cisplatin resistance. As shown in Fig 2B, miR-374a and miR-130a inhibitor significantly enhanced the cytotoxicity of cisplatin treatment compared with that of the control (p<0.05).

### MiR-130a regulate the expression of MDR1 and PTEN in A2780s and A2780/DDP cells

**Fig 3:**
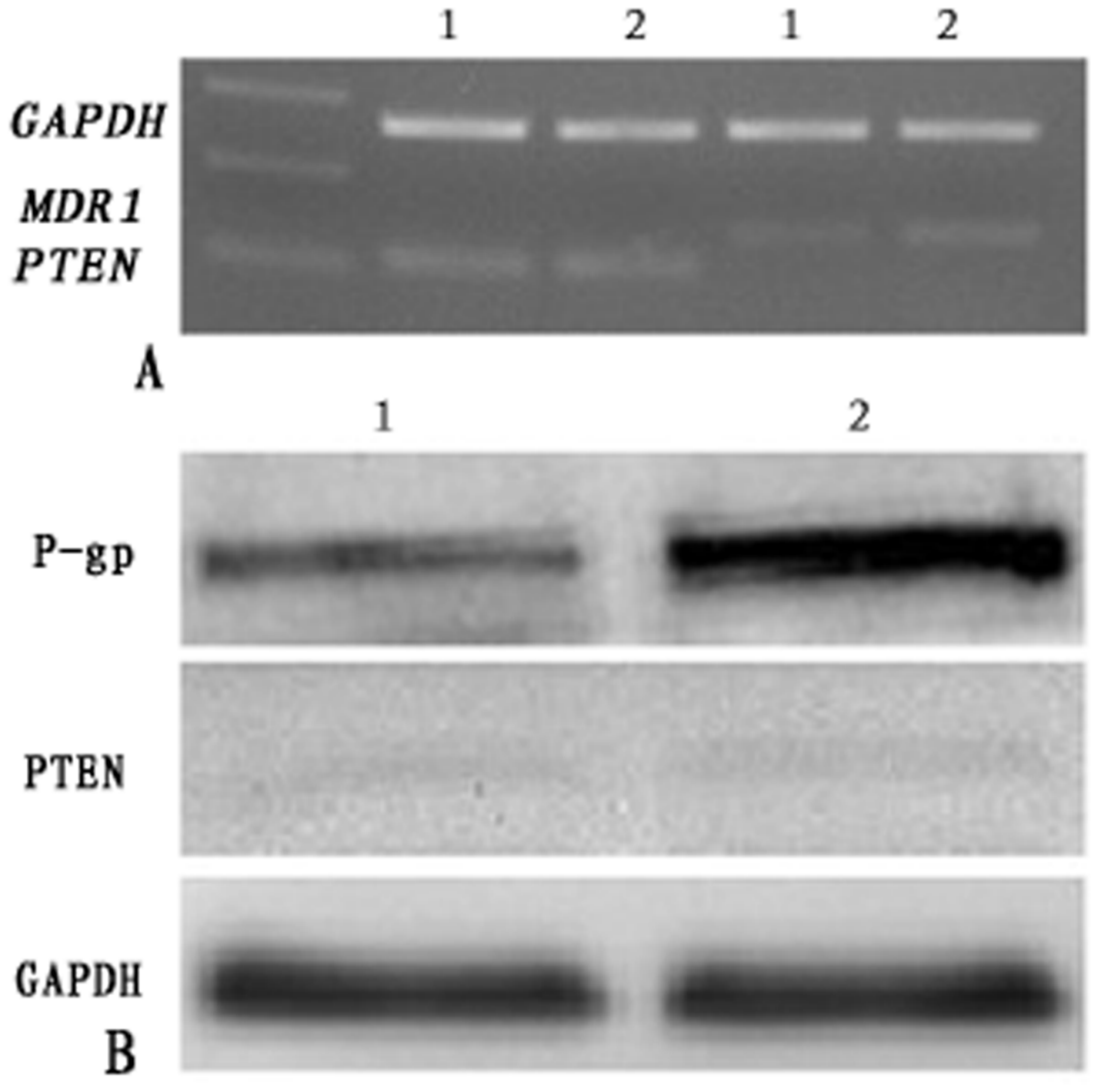
Expression of MDR1、PTEN mRNA and Protein in A2780 and A2780/DDP cells. (A) MDR1 and PTEN mRNA levels in A2780 and A2780/DDP cells. The expression of MDR1 mRNA was overexpressed in A2780/DDP compared with A2780(P<0.05), and the PTEN mRNA level showed no difference(P>0.05). (B) P-gp and PTEN protein expression levels in A2780s and A2780/DDP cells. The expression of P-gp in A2780/DDP cells was higher than that of A2780(P<0.05),and PTEN protein was at a very low levels in A2780 and A2780/DDP cells. (1,2:A2780, A2780/DDP)

We examined the expression of MDR1, PTEN mRNA and protein in A2780 and A2780/DDP cells by RT-PCT and western blot. As shown in Fig 3, the MDR1 mRNA and P-gp level in the A2780/DDP cells was 2.33 and 1.88 times higher than that of in A2780 cells (P<0.05), while the expression of PTEN protein were extremely low in both cell lines and the expression of PTEN mRNA and protein showed no significant difference between them(P>0.05).

**Fig 4:**
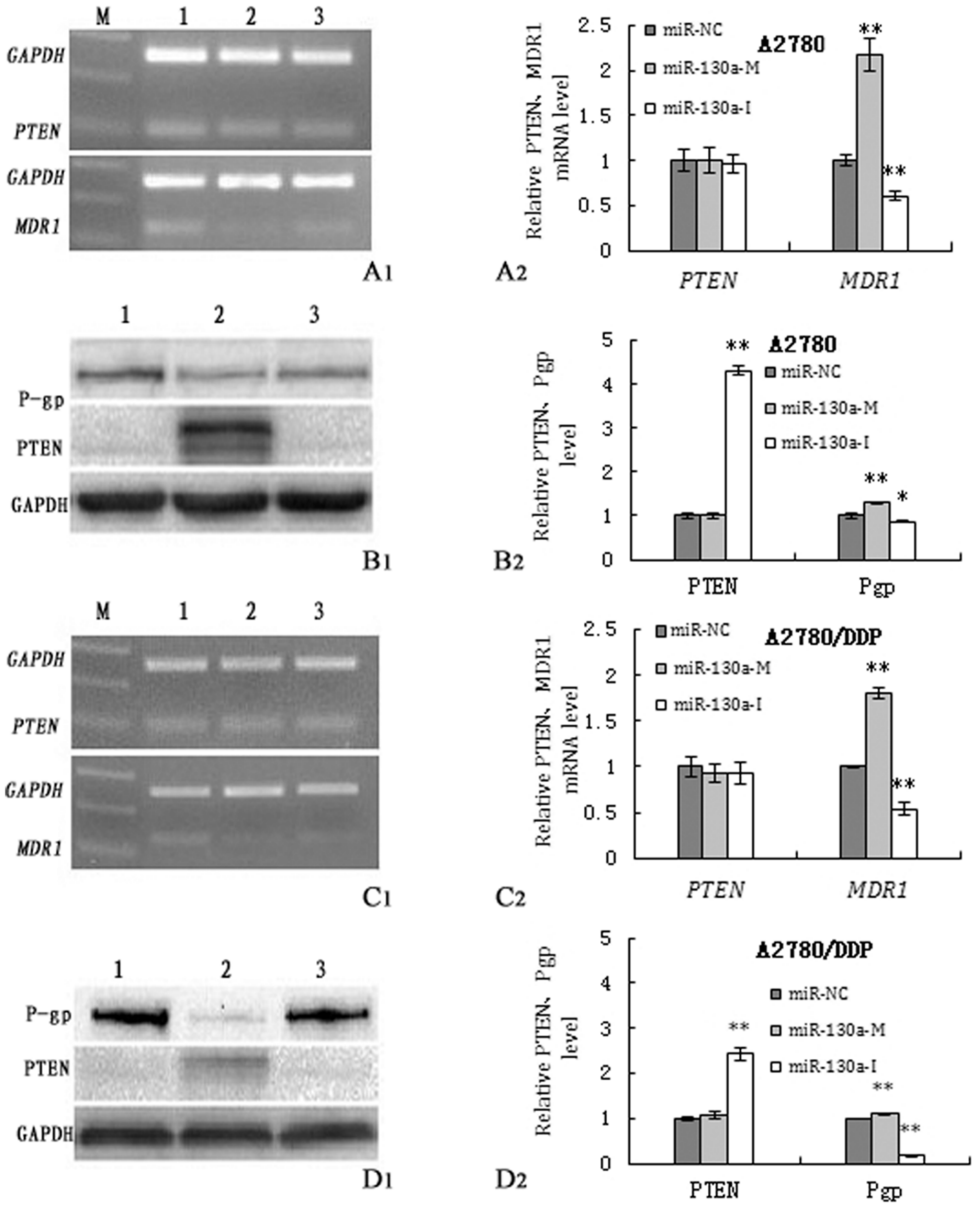
Expression of PTEN, MDR1 mRNA and protein in the transfected cells. (A1,A2): PTEN and MDR1 mRNA in A2780 cells after transfection. The MDR1 mRNA level was upregulated by the miR-130a mimics and downregulated by the miR-130a inhibitor(P<0.01). (B1,B2): P-gp and PTEN protein in A2780 cells after transfection. The expression of PTEN protein was significantly elevated When cells were treated with miR-130a-I, the expression of P-gp were upregulated by miR-130-M and downregulated by miR-130a-I. (C1,C2): PTEN and MDR1 mRNA in A2780/DDP cells after transfection. The regulatory effect of miR-130a on A2780/DDP cells was similar to that of A2780 cells. (D1,D2): P-gp and PTEN protein in A2780/DDP cells after transfection. The effect of miR-130a was similar to that of A2780s cells. (1,2,3:miR-130a-M, miR-130a-I and miR-NC; *p<0.05,**p<0.01)

In order to investigate whether miR-130a could regulate PTEN and MDR1 expression, the corresponding mimics and inhibitors of the two miRNAs were transfected into A2780 and A2780/DDP cells. PTEN, MDR1 mRNA and their protein expression were determined at 48 h after transfection. As shown in Fig 4, miR-130a inhibition increased PTEN protein levels and attenuated the expressio of MDR1 mRNA and P-gp (P<0.05),whereas miR-130a overexpression resulted in the up-regulation of MDR1 mRNA and P-gp expression in A2780 and A2780/DDP cells(P<0.05).The expression of PTEN mRNA didn’t change with the treatment of miR-130a inhibitor and mimics in both cell lines(P>0.05).

In summary, we found 32 miRNAs are differentially expressed in A2780/DDP cells compared to its parental cells by miRNA array. Among them, one down-regulated miRNA (miR-146a) and three up-regulated miRNAs(miR-130a, -374a,-182) were selected for further study, and their expression were validated consistently with miRNA array using qRT-PCR. In the transfection experiment, overexpression of miR-130a and miR-374a were found to decrease the sensitivity of A2780 and A2780/DDP cells to cisplatin, and down-regulating the miR-130a and miR-374a expression exerted the opposite effect. The results of RT-PCR and western blot showed that miR-130a could positively regulate the mRNA and protein expression of MDR1 and negatively regulate the PTEN protein, which may be one of the mechanisms of miR-130a’s role in chemoresistance.

## Discussion

Although platinum-based chemotherapy has greatly improved the prognosis of ovarian cancer, drug-resistance still remains as the main obstacle of successful treatment[3]. Recently, miRNAs were reported to be differentially expressed in drug resistant cancers and could regulate the drug resistance[16,17].

In the present study, we found that 32 miRNAs were differentially expressed in A2780/DDP compared with A2780. Interestingly, some of these miRNAs have been confirmed to be involved in chemoresistance. For instance, miR-27a was up-regulated in A2780/Taxol cells and knockdown of miR-27a enhanced the paclitaxel sensitivity[18]. Takiuchi[19] reported that miR-181 decreased the sensitivity of pancreatic cancer cell to gemcitabine through activating NF-κB by CYLD inhibition. Our previous study also found miR-130a was related to cisplatin-resistance in SKOV3 cells. The data of microarray revealed that in A2780/DDP the expression of miR-146a was extremely low, and miR-130a, -374a, -182 was up-regulated. In addition, miR-130a’s role in cisplatin resistance of A2780 cells and whether miR-146a, -374a, -182 could regulate drug resistance have not yet reported, so we choose these four miRNAs for further study. These four miRNAs expression were verified by qRT-PCR, somewhat suggesting that microarray had a high accuracy.

In the transfection experiments, we found alteration of miR-130a and miR-374a expression could change the degree of cisplatin resistance in A2780 and A2780/DDP. Several researches have shown that miR-130a acted as chemoresistant regulator. Up-regulation of miR-130a could affect the doxorubicin resistance in breast cancer[20], and miR-130a induced the resistance of liver cancer cell to cisplatin by downregulation of tumor suppressor gene RUNX3[21]. In our previous study[15], miR-130a was overexpressed in SKOV_3_/DDP cells, and inhibition of miR-130a could partially restore cisplatin sensitivity, which was consistant with above study.

Currently, a few studies focused on the role of miR-374a in tumor, which pointed out that miR-374a was overexpressed in the osteosarcoma[22] and colon cancer[23]. Besides, miR-374a was involved in the tumor genesis and metastasis of breast cancer by regulating the Wnt/catenin pathway [24]. So far, there are no reports on the role of miR-374a in drug resistance. Here, we found that miR-374a was upregulated in cisplatin-resistant cells, and decreasing its expression could make the cells more sensitive to cisplatin, while upregulating its expression in A2780s had the opposite effect.

Therefore, we considered that over-expression of miR-130a and miR-374a might contribute to the development and regulation of cisplatin resistance in ovarian cancer cells. Inhibiting the expression of miR-130a and miR-374a might be a novel approach for overcoming drug resistance in ovarian cancer. As a result, we also performed RT-PCR and western blot to find the mechanism of miR-130a regulatory effect on drug resistance.

Our study found the expression of MDR1 mRNA and its protein product P-glycoprotein (P-gp) levels in the A2780/DDP was significantly higher than that in the A2780s, suggesting that over-expression of MDR1 gene may be one of mechanism of drug resistance formation in A2780/DDP cells. P-gp is an ATP-dependent membrane pump that transports the drug out of cells, resulting in multidrug resistance[25]. Plenty of evidences indicated that miRNAs were associated with P-gp mediated drug resistance in many cancers. For instance, miR-451 overcame the doxorubicin resistance by downregulating P-gp expression in the doxorubicin resistant breast cancer cell lines MCF-7/ADR [26]. Inhibition of miR-27a enhanced the paclitaxel sensitivity in A2780/Taxol by modulating MDR1/P-glycoprotein expression [18]. We concluded that inhibiting the expression of miR-130a resulted in down-regulating of MDR1 mRNA and P-gp. This result is in accordance with our previous experiments.

We also found that miR-130a negatively regulated the expression of PTEN protein. PTEN protein is a dual specificity phosphatase that can block cytokine-mediated signal transduction pathways, then inhibit cell proliferation, invasion and metastasis and promote apoptosis[27]. In a variety of malignant tumors including colorectal cancer, breast cancer, leukemia, ovarian cancer etc, PTEN gene and protein have different degrees of deletion or down-regulation [27,28]. It has been reported that PTEN protein could contribute to drug sensitivity by suppressing the PI3K/Akt pathway, and serve as target gene of many miRNAs, such as miR-21、miR-22 and miR-222[29,30]. In this experiment, the expression of PTEN protein in A2780s and A2780/DDP cells were extremely low and had no significant difference between these two cell lines. We also inferred that restoration of PTEN expression may be one of mechanisms of chemosensitivity increase in the ovarian cancer cells. However, A2780 and A2780/DDP cells transfected with miR-130a-inhibitor didn’t exert higher cell activity inhibition compared with the control groups under the treatment of low-dose cisplatin (0.2ug/ml and 2ug/ml respectively), we calculated the reason of this phenomenon is that PTEN-mediated cell apoptosis partly rely on the cell damage by cisplatin and they have a synergistic effect. Only a small amount of cells processed to apoptosis or death in this low-dose of cisplatin, and cell growth inhibition mediated by PTEN didn’t have enough ability to make a significant difference between experimental and control groups. Interestingly, We found that miR-130a didn’t regulate PTEN mRNA expression likewise PTEN protein, suggesting that miR-130a may regulate the PTEN gene transcription at post-translational level by incomplete bind to the 3'-untranslated region (3'-UTR) of the PTEN mRNA.

PicTar andTargetScan software predicts that the potential target genes of miR-374a include AKT3, ABI1, PDCD6, ABCC5 and so on. Among them, ABI1 has been proved to be associated with inhibition of cell growth and proliferation[31], which may be one of regulatory mechanisms of miR-374a on cisplatin resistance, however, it remains to be further verified by expriments.

Recently, Xiang et al.[32] reported that miR-152 and miR-185 were significantly downregulated in the SKOV3/DDP and A2780/DDP cells, compared with their sensitive parent line SKOV3 and A2780, and up-regulating the expression of miR-152 or miR-185 increased cisplatin sensitivity of SKOV3/DDP and A2780/DDP cells by suppressing DNA methyltransferase 1 (DNMT1) directly. Inconsistently, miR-152 and miR-185(decreased by 0.4 and 0.6 times in A2780/DDP cells compared with A2780 cells in Xiang’s study) was found to be up- or down-regulated in less than two times in A2780/DDP cells compared with A2780 cells, so these miRNAs were not listed as miRNAs of interest. However, we should be aware that miRNA array is a preliminary screening test whose results need further validation such as by qRT-PCR, and the less than two-fold differentially expressed miRNAs may also have effect on the drug resistance of ovarian cancer.

In conclusion, this study demonstrates that differentially expressed miRNAs in cisplatin-resistant ovarian cancer cells probably play an important role in the development or regulation of cisplatin resistance. Moreover, transfection with miR-130a and miR-374a inhibitors enhanced the cytotoxic effect of cisplatin. The role of miR-130a in drug resistance was achieved at least partially by regulating the expression of P-gp and PTEN protein. PTEN may be a target gene of miR-130a, however, it needs the validation of dual luciferase reports. These results suggest that miR-130a and miR-374a could be promising as novel therapeutic targets for overcoming drug resistance.
